# Association between interleukin-6-174G/C gene polymorphism and asthma severity: exploring the role of total serum IgE, blood eosinophils, and FeNO as markers of type 2 inflammation

**DOI:** 10.1186/s13223-024-00880-0

**Published:** 2024-02-22

**Authors:** Mona Al-Ahmad, Asmaa Ali, Ahmed Maher, Mohammad Z. Haider

**Affiliations:** 1 Department of Microbiology, College of Medicine, Kuwait University, Safat, P.O. Box 24923, 13110 Kuwait City, Kuwait; 2 Department of Laboratory Medicine, School of Medicine, Jiangsu University, Zhenjiang, China; 3 Department of Allergy, Al-Rashed Allergy Center, Ministry of Health, Kuwait City, Kuwait; 4 Department of Pulmonary Medicine, Abbassia Chest Hospital, Ministry of Health, Cairo, Egypt; 5 Department of Pediatrics, College of Medicine, Kuwait University, Kuwait City, Kuwait

**Keywords:** *IL-6*-174G/C gene polymorphism, Mild and severe asthma, Type 2 inflammations

## Abstract

**Background:**

While a connection has been established between serum interleukin-6 (*IL-6*) levels and the *IL-6* gene (− 174G/C) polymorphism in allergic diseases such as asthma, its specific association with severe asthma remains unexplored. This study examined the relationship between the *IL-6* (− 174G/C) gene polymorphism and mild and severe asthma, focusing on its influence on type 2 inflammation.

**Methods:**

Our study comprised 98 patients with mild asthma and 116 with severe asthma. Additionally, we recruited 121 healthy participants to serve as controls for comparative analyses. The *IL-6* gene (− 174G/C) polymorphism was assessed utilizing the polymerase chain reaction-restriction fragment length polymorphism (PCR–RFLP) method.

**Results:**

In our study, the risk of mild asthma exhibited a significant fourfold increase in individuals with the GG genotype pattern compared to healthy controls, yielding an odds ratio (OR) of 4.4 (*p* < 0.001). Conversely, we found no significant correlation between the IL-6 − 174G/C gene polymorphism and severe asthma when compared to the healthy control group. However, a noteworthy pattern emerged when we compared subgroups of mild and severe asthma. The risk of severe asthma increased fivefold in individuals with the GC polymorphism pattern, with an OR of 4.99 (*p* < 0.001), while the likelihood of mild asthma showed a similar fourfold increase with the GG polymorphism pattern, OR = 4.4 (*p* < 0.001). Consequently, we observed a significantly higher frequency of the C allele in patients with severe asthma, whereas the G allele was more prevalent in individuals with mild asthma (*p* = 0.05). Additionally, the correlation between markers of type 2 inflammation and the dominant model of the *IL-6* gene -174G/C polymorphism (CC + CG vs GG) revealed a significant increase in total serum immunoglobulin E (IgE), Blood Eosinophil Counts (BEC), and Fractional Exhaled Nitric Oxide (FeNO) levels in asthmatic patients with the CC + CG gene pattern compared to those with GG, with p-values of 0.04, 0.03, and 0.04, respectively. Furthermore, after adjusting for other risk factors, the likelihood of developing severe asthma increased from fourfold to eightfold, with an OR of 8.12 (*p* = 0.01) with (CC + CG) gene pattern. Other predictors for severe asthma included older age and childhood-onset disease (OR = 1.13 and 19.19, *p* < 0.001). Allergic rhinitis (AR) and nasal polyps (NP) also demonstrated a substantial association with an increased risk of severe asthma, with odds ratios of 5 and 32.29 (*p* = 0.01 and < 0.001), respectively. Additionally, elevated Body Mass Index (BMI), BEC, and FeNO were linked to severe asthma, with ORs of 1.11, 1.00, and 1.04, respectively (*p* = 0.04, 0.05, and 0.001).

**Conclusion:**

This study illuminated the intricate relationship between the IL-6 gene polymorphism, type 2 inflammation markers, and diverse risk factors in shaping asthma severity. As a significant association between the GG polymorphism of the *IL-6* gene (− 174G/C) and mild asthma was found, while possessing at least one C allele, whether in a homozygous (CC) or heterozygous (CG) combination, independently predicts the likelihood of severe asthma.

## Introduction

The *IL-6* gene is located on chromosome 7p21 and has many single nucleotide polymorphisms (SNPs) in the database. However, only three SNPs (− 597 G/A (rs1800797), − 572 G/C (rs1800796), and − 174 G/C (rs1800795) have been extensively studied for their role in different diseases [1]. Individuals may exhibit varying cytokine production levels due to SNPs in crucial regulatory regions, including promoters, introns, and the 5′-UTR and 3′-UTR regulatory regions. These SNPs can impact cytokine expression levels. Genetic polymorphisms in gene-coding regions may also lead to the loss or modification of function in the expressed proteins [2]. The − 174 G/C SNP is located at position 174 within the promoter region of the IL-6 gene [3]. It has been found to influence the expression of IL-6, particularly its G allele, and regulate different inflammatory responses [4–6].

*IL-6* is a typical immune-modulating cytokine with a potential pro-inflammatory ability that stimulates both the innate and adaptive immune systems [7, 8]. The role of this cytokine exceeds that of immune-mediated disease to play a pivotal role in hematopoiesis and the endocrine system [9–11]. Some innate immune cells are responsible for producing IL-6 as B-cells and CD-4 Th cells [7]. Nevertheless, other non-leukocyte cells secrete this cytokine, such as astrocyte cells, fibroblasts, endothelial cells, and some malignant cells [12–16].

Research has shown that the airway epithelium in lung tissue secretes a significant amount of IL-6, along with T-lymphocytes and macrophages. This secretion of IL-6 is believed to be involved in cellular signaling during the development of asthma [17, 18]. The findings suggest that IL-6 can activate the immune system in response to acute phase reactions caused by viral invasion, bacterial infection, and tissue damage. It also plays a role in differentiating T-cells, increasing the production and function of cytokines and mediators, and creating hyper-inflammatory states in severe COVID-19 and septic shock cases [19–24].

New studies have revealed a correlation between heightened levels of IL-6 in the bloodstream and specific allergic conditions, including asthma, allergic rhinitis, and atopic dermatitis [1, 25]. Nevertheless, additional research has indicated that individuals with the IL-6 -174G/C gene polymorphism and corresponding cytokine levels are significantly linked to chronic inflammatory disorders such as coronary heart disease [3], arthritis [26, 27], and metabolic syndromes like diabetes mellitus, hypertension, and obesity [28–31].

Individuals who suffer from allergic diseases such as asthma, allergic rhinitis, and atopic dermatitis experience multiple conditions and frequently possess a shared genetic predisposition. Nevertheless, specific asthma phenotypes deviate from the standard allergy-related procedure, and their pathogenesis varies, often resulting in a more severe ailment [32]. The traditional classification of asthma into extrinsic and intrinsic subtypes also reflects cellular and molecular mechanisms. Hence, extrinsic asthma mainly passes in the allergic pathway and is attributed to Th2 inflammation; however, intrinsic asthma is mainly triggered by other factors, including infections, obesity, exercise, stress, cold, and other causes, and may be passed through different pathogenic mechanisms [33, 34]. However, asthma’s molecular and cellular pathogenesis is complex and overlaps due to different arrays of precipitating factors with dissimilar mechanisms, which could reflect the discrepancy between the traditional classifications of asthma phenotypes. Allergic asthma is initiated in early childhood, continues to adulthood, and is often persistent, with significant variations in disease severity [35, 36].

Several epidemiological studies have found that atopic asthma tends to be less severe than non-atopic asthma [37]. Additionally, patients with mild to moderate asthma tend to have a more robust sensitization response than those with severe asthma [38].

The variations in markers of type 2 inflammations, such as IgE, BEC, and FeNO, differ among various phenotypes/endotypes of asthma. In allergic asthma, a persistent Th2-type inflammatory process is initiated upon exposure to inhaled allergens. Susceptible individuals are more predisposed to activating the airway epithelium and dendritic cells, ultimately leading to the synthesis of specific IgE antibodies [39]. Upon re-exposure to the allergen, cross-linking of FcεRI on tissue mast cells results in immediate bronchoconstriction, eosinophil recruitment, and a late-phase inflammatory response [40]. Detection of specific IgE in the serum through serology or skin-prick testing is a key feature of allergic asthma. Blood eosinophils are typically moderately elevated, and other atopy-related disorders, such as allergic rhinitis and atopic dermatitis, are commonly associated with allergic asthma [41]. The atopic phenotype is genetically determined and influenced by single nucleotide polymorphisms at Th2 genes, and 17q12 loci. While total IgE values overlap between atopic and nonatopic subjects, serum IgE levels closely correlate with asthma risk and airway hyperresponsiveness (AHR), regardless of allergen specificity [42, 43]. High serum IgE levels are more commonly observed in severe and early-onset asthma, and very high levels, greater than 2000 kU L^−1^, correlate with dermatitis severity [44, 45].

On the other hand, around 25–30% of people with asthma experience the “nonallergic” phenotype, which is characterized by asthma onset in adulthood without any known cause. Within this phenotype, there is a subset called intrinsic asthma that is different from allergic asthma because there are no detectable specific IgE antibodies in the blood, and skin-prick tests for common allergens are negative [46]. Clinical features of intrinsic asthma include a later onset, no family history of asthma, higher prevalence in women, and often being associated with chronic rhinosinusitis with nasal polyps, which can lead to a more severe form of the disease [47].

People with nonallergic asthma may have higher levels of total serum IgE than healthy individuals [43]. Although there are differences between allergic and intrinsic asthma, there is some overlap in immune cell infiltration and local IgE synthesis [43]. However, the allergen specificity of the IgE remains unknown. A subset of intrinsic asthma known as “hyper-eosinophilic asthma,” which occurs later in life and is not caused by allergies; this type is associated with nasal polyps and high levels of eosinophils in the blood (over 1000 μL–1 or over 500 μL–1 when treated with oral corticotherapy). Biological therapy that targets IL-5, such as mepolizumab and reslizumab, are effective treatments for this subtype [48]. Severe cases are characterized by high levels of eosinophils in both blood and sputum, more commonly found in males, airflow limitation, elevated FeNO, and frequent severe exacerbations [49]. Isolated sputum eosinophilia is less linked with poor control but is associated with higher serum IgE levels [43, 50]. There is no identified genetic predisposition, but IgE responses to enterotoxin-producing S. aureus colonizing the upper airways are widespread [51]. Staphylococcal superantigens can cause polyclonal T- and B-cell activation [52]. Anti-IgE therapy has shown to be beneficial in asthma with nasal polyps, regardless of atopic status, supporting the role of IgE in hypereosinophilic nonallergic asthma [53].

Moreover, a distinct subgroup without evidence of Th2 eosinophilic inflammation was reported and characterized by severe corticosteroid resistance shares features with chronic obstructive pulmonary disease. This phenotype may be more accurately termed “type 2-low” asthma, given the involvement of alternative producers like ILC2s in “type 2” cytokine production [43]. The role of IgE in this phenotype is unclear, but IL-17, IL-33, and ADAM8 have been implicated [54].

To date, there is a notable gap in research addressing the inflammatory implications of *IL-6* gene -174G/C polymorphism in adult asthma, particularly within Kuwait and the Gulf region. This study aims to fill this gap by investigating the association between the IL-6 gene − 174G/C polymorphism and asthma severity in adults, distinguishing between mild and severe asthma and assessing its influence on type 2 inflammation while focusing on total serum IgE, BEC and FeNO levels as markers of type 2 inflammations.

## Patients and methods

### Patients and study design

This case–control study was conducted et al.-Rashed Allergy Center, Kuwait, from December 2022 to April 2023. The study enrolled 98 mild and 116 severe asthma patients based on clinical diagnosis and reversibility of FEV1% [55]. The study also included 121 healthy controls for comparison. Patients with mild asthma were characterized by infrequent symptoms and relatively well-preserved lung function, which minimally impacted their daily activities. In contrast, severe asthma manifested persistent symptoms, frequent exacerbations, frequent use of corticosteroid courses, and significant lung function limitations, often requiring intensive treatment and medical intervention [55, 56]

### Ethics approval and consent to participate

Ethical approval has been obtained from Kuwait University and the Ministry of Health, Kuwait (Research project number MI02/22, 2022/2010), and to ensure that the research is conducted ethically and in compliance with the internationally recognized standards, Helsinki Declaration protocol were applied.

Informed consent has been obtained from all participants involved in the study and their legal guardians to ensure that they are fully aware of the nature and purpose of the research; they have given their informed consent voluntarily to participate.

### Sample size

The sample size required for this study was calculated using Minitab 17.1.0.0 for Windows software (Minitab Inc., 2013, Pennsylvania, USA). To achieve 80% power, we considered an odds ratio of 2, a disease prevalence of about 1%, a minor allele frequency of 5%, complete linkage disequilibrium (LD), and a 5% error rate in an allelic test. This study’s minimum total sample size was 248, with a 1:1 case/control ratio. Additionally, to ensure a robust representation of the subgroup of cases with varying degrees of asthma severity (mild and severe), we included an equal number of mild asthma cases, assuming a case: case ratio similar to the control: case ratio, to achieve 80% study power.

### Sample collection

In a sterile setting, 10 mL of venous blood was obtained from each participant via a plastic syringe. The blood was then split into two tubes—one without anticoagulant and the other with EDTA to aid DNA extraction. After centrifugation at 4000 g for 10 min, the serum, plasma, and buffy coat with leukocytes were separated. The samples were then frozen at − 20 °C for DNA extraction. Genomic DNA was extracted using the QIAamp Blood Kit (QIAGEN, Germany) following the manufacturer's protocol. The purity and quantity of the extracted DNA were evaluated using a Nanodrop 8000 spectrophotometer (Thermo-Scientific, Delaware, USA), measuring absorbance at wavelengths 260 and 280 nm. DNA purity was assessed by the A260/A280 ratio, with the target range set between 1.8 and 2.0. To estimate DNA concentration, the optical density (O.D.) at 260 nm was measured, and the concentration (mg/ml) was calculated using the formula: Concentration = O.D. 260 × 50 x dilution factor (× 100). The final DNA concentration was directly determined using the Nanodrop 8000 spectrophotometer software, falling within the optimal range of 107–552 ng/μL.

### Genotyping

The genotypes for *IL6* gene (− 174G/C; rs1800795) polymorphism were identified by PCR–RFLP (polymerase chain reaction-restriction fragment length polymorphism). The details about primers and PCR method used are given below:

Forward primer: 5ʹ-GGAGTCACACACTCCACCT-3ʹ,

Reverse primer: 5ʹ-GTGGGGCTGATTGGAAACC-3ʹ.

The PCR reactions were carried out in a total volume of 25 ml containing 100 ng of genomic DNA, 10 pmol of each primer, 2 mM MgCl2, 0.2 mM deoxynucleotides (dNTPs), 1 × buffer, and 2U of Taq DNA polymerase. For IL-6 gene (− 174G/C) polymorphism, the amplification was performed for 35 cycles with an initial denaturation step at 94 °C for 5 min followed by the PCR cycles of 94 °C for 1 min, annealing at 65 °C for 1 min, 72 °C for 1 min and then an extension step at 72 °C for 10 min. The polymorphism was identified by cleavage of the PCR products with restriction enzyme *Sfa*N1. The GàC change at position 174 creates a restriction site for this enzyme and consequently, G-allele produces an un-cleaved 532 bp product while the C-allele yields two products of 474 and 58 bp, respectively upon digestion of the PCR products with *Sfa*N1.The DNA cleavage fragments were resolved by electrophoresis on a 3% agarose gel and were visualized under UV light after Ethidium bromide staining.

### Statistical analysis

Demographic data of patients and control groups were collected in an Excel sheet, along with the genotype of each study participant. Minitab for Windows (Minitab Inc, 2013, version 17.1.0.0, Pennsylvania, USA) was used for statistical analysis. The data is represented as mean and standard deviation for numerical data and number (%) for categorical data. Comparison between two means was performed using an independent t-test and between two frequencies through a Chi-square test. Logistic regression analysis with adjusted and non-adjusted methods was applied to find the predictive ability of *IL-6 -174G/C* gene polymorphism and severe asthma. All tests were two-sided, and p < 0.05 was considered significant.

## Results

In Table 1, a comparison of demographic characteristics and the frequency of IL-6 -174G/C gene polymorphism was conducted between control subjects and asthma subgroups. The patients with severe asthma exhibited a significantly higher age (52.8 ± 11.5 years) compared to the control (37.5 ± 16.1 years) and mild asthma subgroups (38.3 ± 16.6 years), with a p-value of < 0.001. However, the three groups were matched with respect to gender and BMI.

**Table 1:** Comparing the demographic characters and the frequency of *IL-6* gene (-174G/C) polymorphism in control versus mild and severe asthma subgroups

Factors	Control (n = 121)		Mild asthma (n = 98)		Severe asthma (n = 116)		OR	95% CI	*P1*	OR	95% CI	*P2*	OR	95% CI	*P3*
	Mean	SD	Mean	SD	Mean	SD									
Age (Years)	37.5	16.1	38.3	16.6	52.8	11.5			0.73			** < 0.001**^**†**^			** < 0.001**^**†**^
Female-sex	83	68.6	66	67.35	80	68.97			0.84			0.95			0.81
BMI	29.91	8.92	30.19	6.95	31.46	5.75			0.62			0.09			0.15

*IL-6* polymorphisms	N	%	N	%	N	%	OR	95% CI		OR	95% CI		OR	95% CI	
CC	77	63.64	56	57.14	62	53.45	1.313	(0.761–2.264)	0.32	1.524	(0.906–2.563)	0.11	0.898	(0.523–1.541)	0.69
GC	32	26.45	10	10.2	42	36.21	0.316	(0.147–0.682)	0.002*	1.579	(0.907–2.746)	0.12	4.995	(2.346–10.635)	< 0.001*
GG	12	9.92	32	32.65	12	10.34	4.404	(2.121–9.143)	< 0.001*	1.048	(0.451–2.438)	0.91	4.4	(2.121–9.128)	< 0.001*
C allele	186/242	76.85	122/196	62.24	166/232	71.55	0.41	(0.29, 0.59)	0.001*	0.57	(0.435, 2.834)	0.54	1.53	(1.017–2.289)	0.05*
G allele	56/242	23.14	74/196	37.75	66/232	28.44	1.67	(1.15, 2.44)	0.006*	1.13	(0.874–1.996)	0.22	0.65	(0.437–0.984)	0.05*

Dominant pattern	N	%	N	%	N	%	OR	95% CI		OR	95% CI		OR	95% CI	
CC + CG	109	90.08	66	67.35	104	89.66	4.404	(2.121–9.143)	< 0.001*	1.04	(0.451–2.438)	0.91	4.4	(2.121–9.128)	< 0.001*
GG	12	9.92	32	32.65	12	10.34	Reference			Reference					

Recessive pattern	N	%	N	%	N	%	OR	95% CI		OR	95% CI		OR	95% CI	
CC	77	63.64	56	57.14	62	53.45	Reference		0.32	Reference		0.11			
CG + GG	44	36.36	42	42.86	54	46.55	1.313	(0.761–2.264)		1.52	(0.906–2.563)		0.898	(0.523–1.541)	0.65

*N* number, *SD* standard deviation, *OR* odd ratio, *CI* Confidence interval

Numerical data represented as mean and standard deviation and categorical data as number and percentage, p1: Control versus mild asthma, p2: control versus severe asthma, p3, mild asthma versus severe asthma, the test of significant: ^†^: Independent t-test, *: Chi square test

Text with Bold represents a significant p value; *p* < 0.05

Moreover, the polymorphism pattern of *IL-6* -174G/C gene subgroup analysis showed that the risk of mild asthma increased fourfold with the GG genotype pattern compared to healthy control, OR = 4.4, *p* < 0.001. However, the GC pattern showed a protective ability against mild asthma, OR = 0.31, *p* = 0.002. Additionally, the frequency of the G allele was significantly higher in mild asthmatic patients, OR = 1.67, *p* = 0.006. Conversely, the frequency of the C allele was significantly observed in the control subject, OR = 0.41, *p* = 0.001. The dominant model of *IL-6* -174G/C gene (CC + CG vs GG) showed that the risk of mild asthma decreased fourfold, OR = 4.4, *p* < 0.001, while the recessive model (CC Vs CG + GG) showed insignificant association, *p* = 0.32. On the other hand, no correlation was found between the *IL-6* -174G/C gene polymorphism and severe asthma compared to healthy controls. However, in comparing mild and severe asthma subgroups, the risk of severe asthma increased fivefold with the GC polymorphism pattern, OR = 4.99, *p* < 0.001, while the likelihood of mild asthma increased fourfold with the GG polymorphism pattern, OR = 4.4, *p* < 0.001. Thus, the frequency of the C allele was significantly higher in patients with severe asthma, while the G allele was higher in mild asthma patients, *p* = 0.05. The use of dominant model of *IL-6* gene − 174G/C polymorphism (CC + CG vs GG) showed that (CC + CG) increased the likelihood of severe asthma fourfold, OR = 4.4, *p* < 0.001. Yet, in the recessive model (CC vs CG + GG) revealed an insignificant association, *p* = 0.65.

In Table 2, a comparison between mild and severe asthma regarding clinical features and markers of type 2 inflammation revealed notable differences. Patients with severe asthma exhibited significantly higher prevalence of comorbidities, specifically allergic rhinitis (AR) and nasal polyp (NP), at 73.28% and 49.14%, respectively, with a p-value of < 0.001. Additionally, levels of total serum IgE, BEC, and FeNo were significantly elevated, with p-values of < 0.001, 0.001, and 0.002, respectively. Conversely, the FEV1% predicted was significantly lower in the severe asthma group, demonstrating a p-value of 0.03. Furthermore, the frequency of exacerbations requiring an oral corticosteroid (OCS) course was significantly higher in the severe asthma group, with a p-value of 0.001. Additionally, the correlation between markers of type 2 inflammation and the dominant model of the *IL-6* gene -174G/C polymorphism (CC + CG vs GG) was presented in Table 3. The results revealed a significant increase in total serum IgE, BEC, and FeNO levels in asthmatic patients with the CC + CG gene pattern compared to those with GG, with p-values of 0.04, 0.03, and 0.04, respectively.

**Table 2:** Characteristics and type 2 biomarkers in asthma patients

Factors	Mild asthma (n = 98)		Severe asthma (n = 116)		*p*
Smoking	N	%	N	%	
Smoker	10	10.2	5	4.31	0.19^c^
Ex-smoker	3	3.06	6	5.17	
Non-smoker	85	86.73	105	90.52	

Asthma onset	N	%	N	%	
Adulthood	61	62.24	84	72.41	0.11^c^
Childhood	37	37.76	32	27.59	

Associated morbidity	N	%	N	%	
DM	6	6.12	9	7.76	0.63^c^
AR	46	46.94	85	73.28	** < 0.001**^c^
NP	8	8.16	57	49.14	** < 0.001**^c^
Eczema	7	7.14	8	6.9	**0.98**^c^

Evaluation	Mean/median	SD/IQR	Mean/median	SD/IQR	
BMI	30.19	6.95	31.46	5.75	0.15^**a**^
IgE	114	(35–298)	275	(130–566)	** < 0.001**^**b**^
Blood Eosinophil Counts	0.21	(0.1–0.45)	0.41	(0.19–0.73)	**0.001**^**b**^
FeNO	14.5	(8–25)	23	(11–45)	**0.002**^**b**^
FEV1% predicted	80.395	(69–111)	58.25	(21–109)	**0.03**^**b**^

	N	%	N	%	
Exacerbation one year before	6	6.12	32	27.59	**0.001**^c^
OCS course number	3	3.06	25	21.5	**0.001**^c^

Numerical data represented as mean and standard deviation or median and inter quartile range, and categorical data as number and percentage

*N* number, *SD* standard deviation, *IQR* Inter quartile range, *OR* odd ratio, *CI* Confidence interval, *BMI* body mass index, *IgE* immunoglobulin E, *FeNO* Fractional expired nitric oxide, *DM* diabetes mellitus, *AR* allergic rhinitis, *NP* nasal polyp

^a^Independent t-test

^b^Mann Whitney test

^c^Chi square test

Text in Bold represents a significant p value; *p* < 0.05

**Table 3:** Type 2 inflammatory markers in correlation with dominant model of IL-6 gene (-174G/C) polymorphism

Factors	GG (n = 45)		CC + CG (n = 169)		p
	Mean/median	SD/IQR	Mean/median	SD/IQR	
Age (Years)	40.1	18.7	47.5	14.8	**0.01**^**a**^
Female-sex	33	73.33	113	66.86	0.41^c^
BMI	30.96	7.27	30.86	6.1	0.93^c^
IgE	123	(36–400)	191.1	(69–522)	**0.04**^**b**^
BE	0.2	(0.1–0.5)	0.3	(0.2–0.7)	**0.03**^**b**^
FeNO	13	(4–22)	17	(9–33)	**0.04**^**b**^

Numerical data represented as mean and standard deviation or median and inter quartile range, and categorical data as number and percentage

*N* number, *SD* standard deviation, *IQR* Inter quartile range, *BMI* body mass index, *IgE* immunoglobulin E, *FeNO* Fractional expired nitric oxide

^a^Independent t-test

^b^Mann Whitney test

^c^Chi square test

Text in Bold represents a significant p value; *p* < 0.05

Moreover, the predictors of severe asthma are outlined in Table 4, with the prominent *IL-6* gene -174GC polymorphism (CC + CG) model emerging as an independent predictor. Upon adjusting for other risk factors, the likelihood of severe asthma significantly increased, ranging from fourfold to eightfold, presenting an odds ratio (OR) of 8.12 with a p-value of 0.01. Other independent predictors for severe asthma encompassed older age and childhood-onset disease, yielding odds ratios of 1.13 and 19.19, respectively, with p-values less than 0.001. Additionally, AR and NP substantially heightened the risk of severe asthma, with odds ratios of 5 and 32.29 (p < 0.01 and < 0.001, respectively). Furthermore, elevated BMI, BEC, and FeNO levels were also associated with severe asthma, exhibiting odds ratios of 1.11, 1.00, and 1.04, and corresponding p-values of 0.04, 0.05, and 0.001, respectively.

**Table 4:** Predictors of severe asthma

Factors	OR	95% CI	*p*
Un-adjusted model			
Dominant (CC + GC)	4.40	(2.1209, 9.1283)	** < 0.001**
Adjusted model			
Age	1.13	(1.0727, 1.1999)	** < 0.001**
Sex (male)	1.32	(0.2986, 5.8428)	0.71
Asthma onset (Childhood)	19.19	(3.3314, 110.5572)	** < 0.001**
AR	5.00	(1.2885, 19.4386)	**0.01**
NP	32.29	(6.2848, 165.8691)	** < 0.001**
BMI	1.11	(1.0051, 1.2214)	**0.04**
IgE	1.00	(0.9999, 1.0033)	**0.05**
FeNO	1.04	(1.0168, 1.0729)	**0.001**
Dominant (CC + GC)	8.12	(1.4630, 45.0226)	**0.01**

The test of fitness: Hosmer–Lemeshow test, X2 = 7.77, p = 0.45, the test of significance: Logistic regression models with backward elimination methods

*OR* odd ratio, *CI* Confidence interval, *BMI* body mass index, *IgE* immunoglobulin E, *FeNO* Fractional expired nitric oxide, *DM* diabetes mellitus, *AR* allergic rhinitis, *NP* nasal polyp

Text in Bold represents a significant p value; *p* < 0.05

## Discussion

Various research studies have attempted to examine the role of genetic factors in the development and progression of severe asthma [57]. However, a comprehensive understanding of this topic still needs to be improved. Given the diversity of asthma, various endo/phenotypes have arisen, each indicative of a unique pathogenic process [58]. The main objective of genetic investigations in a multifaceted condition such as asthma is to pinpoint the link between a cluster of genes or their variations and the onset or progression of the disease toward a severe state [59].

This study aimed to explore the possible association between the *IL-6* gene (− 174G/C) polymorphism and adult patients with bronchial asthma, emphasizing assessing its correlation with the disease severity. The finding revealed that in comparing mild asthma with control subjects, the risk of mild asthma increased four and two-fold in homogenous (GG) and allele comparison (G), respectively, and reduced in dominant (CC + GC), heterogeneous (GC), and allele comparison (C), respectively. However, in comparing mild asthma with severe one, the risk of severe asthma increased four, five, and two-fold in dominant (CC + GC), heterogeneous (GC), and allele (C) comparisons, respectively, and reduced in homogeneous (GG) comparisons.

In previous literature [60, 61], the relationship between the *IL-6* gene (− 174G/C) polymorphism and bronchial asthma remains inconclusive. A comprehensive meta-analysis study examined the potential correlation between the *IL-6* gene (− 174G/C) polymorphism and allergic diseases in a sample of 1282 patients with allergic ailments and 1902 control subjects [1]. The findings revealed that, in the general population, there was no notable link between the *IL-6* gene − 174G/C polymorphism and the overall risk of allergic diseases. However, trends were contrasted when the analysis was based on ethnicity. Among Caucasians, the *IL-6* gene (− 174G/C) polymorphism was correlated with a reduced risk of overall allergic diseases in dominant, allele, and heterozygote comparisons. Conversely, in Asian populations, the *IL-6* gene (− 174G/C) polymorphism was associated with an increased risk of overall allergic diseases in dominant, allele, and homozygote comparisons. Additionally, the study found that the *IL-6* (− 174G/C) variant significantly correlates with a lower risk of childhood allergic diseases across different age groups, observed in multiple comparisons, including dominant, allele, heterozygote, and homozygote comparisons. Furthermore, the homozygote comparison remarkably reduced the risk of allergic diseases.

Investigating a connection between *IL-6* gene variations and specific allergic conditions is crucial due to the contribution of various factors and epigenetic influences on different allergic diseases. This approach is necessary to assess the effectiveness of the study for each category individually. A more extensive analysis included 746 asthmatic patients and 1145 controls and investigated the link between the *IL-6* gene (− 174G/C) polymorphism variant and asthma risk [1]. The results reported a significant association between *IL-6* gene (− 174G/C) polymorphism and asthma risk, specifically in the recessive and homozygote comparisons. Moreover, a subgroup analysis by ethnicity and age revealed that among Caucasians, the* IL-6* gene (− 174G/C) polymorphism decreased asthma risk in both the recessive and homozygote comparisons. However, in children, it reduced asthma risk in the dominant, heterozygous, and homozygous comparisons. A similar trend was observed in adults under the homozygote comparison [62]. In another meta-analysis, the data also discovered that CC genotype carriers exhibited a protective effect against asthma in a study of Caucasian populations [60, 62]. However, no evidence was found to support this in the Asian population [63]. Nevertheless, further analysis revealed that the CC genotype may offer protection against asthma in adults [60, 64]. These findings suggest that the *IL-6* gene (− 174G/C) polymorphism may play a role in asthma pathogenesis by altering transcriptional regulation and serum IL-6 levels [60, 64, 65].

On the other hand, other studies have refuted any association between IL-6 cytokines and its gene -174G/C polymorphism with bronchial asthma and have considered IL-6 a product of airway inflammation [66, 67].

In a different aspect, a supportive finding came with Noss et al., the study revealed the relationship between genetic variation, protein expression, and IL-6 regulation in rheumatoid arthritis [68]. They identified a specific association between the *IL-6* proximal promoter polymorphism rs1800795 and heightened IL-6 production in fibroblasts; the highest IL-6 levels were linked to a genetic variant (minor allele C), thus emphasizing the importance of examining diverse cell types. The significance of his study lies in its ability to offer valuable insights for interpreting genetic associations and unraveling the complexities of gene regulation in chronic inflammatory diseases such as rheumatoid arthritis.

In a smaller study, patients with infantile asthma were shown to have a strong correlation with *IL-6* gene (-174G/C) polymorphism; the likelihood of asthma increased up to fourfold, especially in the atopic phenotype [69]. The inconsistency among genetic studies regarding asthma may be attributed to the complex nature of its origin and the diverse forms it can take.

Our results demonstrate that patients with severe asthma were generally older patients than in those with mild asthma. Notably, many of these patients also suffered from associated AR and NP. Additionally, total serum IgE and BEC were significantly elevated, along with FeNO; these pointed to the presence of type 2 inflammation in patients with severe asthma. Moreover, this study showed a connection between the dominant pattern of the IL-6 gene (-174G/C) and markers of type 2 inflammation. Specifically, it demonstrated that the dominant variation (CC + CG) is associated with increased levels of total serum IgE, BEC, and FeNO compared to the GG variation. Additionally, the study found that predictors of severe asthma, besides the dominant variation of* IL-6* gene (-174G/C) polymorphism, were being older with childhood-onset disease. We also report that the likelihood of severe asthma increased two-fold every year and 19-fold in patients with childhood-onset disease. In addition, the AR and NP increased the risk of severe asthma five and 32-fold.

Individuals with severe asthma often experience persistent and challenging symptoms that are difficult to manage [70]. Unlike typical asthma cases, this refractory condition is characterized by an inadequate response to traditional treatments and heightened, prolonged symptoms [71]. Asthma involves lung inflammation, airway hyper-responsiveness, airway remodeling, and excessive mucus production, all contributing to airflow issues. It is not one disease but a combination of variations in these features [72]. Severe asthma significantly impairs patients' quality of life and managing the disease can be daunting [73]. Despite extensive research to understand its causes and treatment responses, many aspects of severe asthma are still unknown. Our patients with severe asthma exhibited steroid resistance and required additional biological therapy to control the disease, in addition to managing comorbidities such as nasal polyps and allergic rhinitis. A recent study in Kuwait found a high prevalence of asthma in patients with nasal polyps [74], and the GG genotype was significantly associated with both nasal polyps and bronchial asthma [75]. However, the study did not determine the severity of asthma. Another study found that GG genotype was significantly reported in 67% of patients with NP [76]. The development of polyps in the nasal cavity is attributed mainly to chronic inflammation, closely resembling the pathogenesis of severe asthma. Various types of inflammatory cells, including eosinophils, neutrophils, plasma cells, lymphocytes, and mast cells, contribute to this process [77]. The inflammatory pathways Th1 and Th2 are activated by signaling molecules, such as IL-6, further exacerbating the inflammatory response [78].

Unlike NP, research findings indicate that possessing the CC genotype of the *IL-6* gene (-174G/C) polymorphism may increase the likelihood of developing allergic rhinitis two-fold in the Chinese population [62]. Likewise, studies conducted in Iran suggest that the *IL-6* gene (− 174G/C) polymorphism could contribute to the onset of AR [67].

While patients with mild and severe asthma were initially matched in this study for BMI during univariate analysis, the multivariate analysis revealed that the probability of severe asthma doubled with every one-point increase in BMI. Surprisingly, this factor did not correlate with the dominant polymorphism pattern of the *IL-6* gene (− 174G/C). In contrast, a study investigating specific genetic variations in the *IL6* promoter, including − 174 G/C and − 572 G/C polymorphisms, found associations with insulin resistance, dyslipidemia, and increased serum insulin release [79]. Composite genotype and haplotype analyses involving all three *IL6* promoter variants were linked to type 2 diabetes, obesity, and metabolic syndrome in Caucasians. These findings align with a parallel study in children [80], which explored the association between the G-174C polymorphism of the *IL-6* gene and obesity as well as the incidence of metabolic syndrome (MetS). Interestingly, carriers of the C allele, in both homozygotic and heterozygotic genotypes, were more prevalent among obese children. However, despite a 33% incidence of MetS in the obese group, the analysis did not establish a significant association between the 174G > C polymorphism and either obesity or the occurrence of MetS in children. This discrepancy suggests the presence of other influencing factors beyond the *IL-6* 174G > C gene polymorphism in patients with inflammatory diseases like metabolic syndrome or asthma.

Our study has certain limitations that need to be acknowledged. Firstly, the patients and control subjects were all selected from a single center, which might have caused a selection bias. However, we took measures to address this issue by carefully matching the control group with the patients, especially those with mild asthma. Secondly, we only investigated polymorphisms within the *IL-6* gene without examining other genetic variants in asthma development.

In conclusion, patients with severe asthma were notably older. They had significant comorbidities such as AR and NP, accompanied by high levels of BEC, total serum IgE, and FeNO. The dominant model (CC + GC) of the *IL-6* gene (-174G/C) polymorphism was significantly associated with severe asthma and influencing type 2 inflammatory markers.

## Data Availability

The datasets used during the current study are available from the corresponding author on reasonable request.
